# Differences in Perceptions of Gun-Related Safety by Race and Gun Ownership in the United States

**DOI:** 10.1017/jme.2023.38

**Published:** 2023

**Authors:** Julie A. Ward, Mudia Uzzi, Talib Hudson, Daniel W. Webster, Cassandra K. Crifasi

**Affiliations:** 1: CENTER FOR GUN VIOLENCE SOLUTIONS, JOHNS HOPKINS BLOOMBERG SCHOOL OF PUBLIC HEALTH, BALTIMORE, MD, USA; 2: THE NEW HOOD, THE NEW SCHOOL, NEW YORK, NY, USA

**Keywords:** Public Opinion, Firearms, Personal Safety, Gun-Related Beliefs, Health Status Disparities, Health Equity

## Abstract

Motivated by disparities in gun violence, sharp increases in gun ownership, and a changing gun policy landscape, we conducted a nationally representative survey of U.S. adults (n=2,778) in 2021 to compare safety-related views of white, Black, and Hispanic gun owners and non-owners. Black gun owners were most aware of homicide disparities and least expecting of personal safety improvements from gun ownership or more permissive gun carrying. Non-owner views differed. Health equity and policy opportunities are discussed.

In 2020, nearly 20,000 people were killed by interpersonal acts of gun violence in the United States, including through community violence, police violence, and intimate partner violence.^1^ This represents an age-adjusted, single-year increase of 35%, despite a greater than 5% decline in overall crime during the same period.^2^ Gun violence and the psychological burden imposed by gun violence disproportionately affect minoritized communities. Nationally, the U.S. gun homicide rate (per 100,000 people) in 2020 was 26.6 for people identified as Non-Hispanic Black, compared to 2.2 for those identified as Non-Hispanic white and 4.5 for Hispanic individuals of any race.^3^ Additionally, Black men are estimated to be 2.5 times more likely to be killed by police than white men; Latino men are 1.3-1.4 times more likely.^4^ For women, the highest rates of homicide in the U.S. are among Black or Native American women.^5^ Surveys by the Pew Research Center conducted since 2018 have consistently found that a large majority (78-82%) of Black Americans consider gun violence to be “a very big problem” in the United States. In comparison, 42-47% of white Americans and 57-59% of Hispanic Americans responded the same, indicative of disparities in both gun violence and perceptions of gun violence.^6^

One response to violence-related concerns could be to purchase a gun, as millions of Americans did in 2020.^7^ For more than 90% of gun buyers in 2020, protection from other people was a primary motivator of the purchase.^8^ However, gun possession also poses risks, by introducing highly lethal means for suicide, increasing the potential lethality of violence in the home, raising the possibility of unintentional injury, or amplifying perceived aggression and threat assessed by police or other armed individuals.^9^ Research suggests that gun acquisition, though often motivated by personal safety interests, frequently leads to incomplete resolution of safety-related concerns.^10^

Perceptions of risk for victimization, beliefs about guns, and feelings of personal safety are likely to be interrelated and informed by racialized experiences of violence and gun ownership. However, the skewed racial distribution of gun ownership often prohibits researchers of gun-related risk perception from examining potentially important social nuance stemming from both race and gun ownership. The objectives of this study were to 1) assess perceptions of disparities in gun violence victimization alongside views on the personal safety consequences of gun possession and carrying, and 2) assess differences in these views by racial subgroups of gun owners and non-owners using a nationally representative sample of U.S. adults.

Perceptions of risk for victimization, beliefs about guns, and feelings of personal safety are likely to be interrelated and informed by racialized experiences of violence and gun ownership.^11^ However, the skewed racial distribution of gun ownership often prohibits researchers of gun-related risk perception from examining potentially important social nuance stemming from both race and gun ownership.^12^ The objectives of this study were to 1) assess perceptions of disparities in gun violence victimization alongside views on the personal safety consequences of gun possession and carrying, and 2) assess differences in these views by racial subgroups of gun owners and non-owners using a nationally representative sample of U.S. adults.

## Methods

### Data

The National Survey of Gun Policy has been administered every two years since 2013, using NORC’s Amerispeak Panel. The 2021 survey was fielded January 4-20, 2021. The biennial survey consists of a core module of gun policy questions repeated over time.^13^ In 2021, a supplemental module containing items related to public safety and violence prevention was also included.^14^ NORC’s Amerispeak Panel is a nationwide probability-based sample of 97% of U.S. households, drawn from the U.S. Postal Service Delivery Sequence File with supplementation by NORC field surveillance for improved rural area coverage. The panel’s recruitment rate is 34%. Survey participation is encouraged through modest cash incentives, with the typical panel member participating in 2-3 surveys per month.^15^ For this survey, invited panelists were 18 years or older and Spanish- or English-speaking. Respondents could complete the survey online or by telephone in their preferred language. To enable comparisons of underrepresented groups, we oversampled Black Americans, Hispanic Americans, and gun owners. Respondents self-identified their race or ethnicity as “White, non-Hispanic;” “Black, non-Hispanic;” or “Hispanic.” “Other, non-Hispanic;” “Multiple, non-Hispanic;” and “Asian, non-Hispanic” were additional response options that are not discussed in this paper due to insufficient sample size. Gun owners were identified through affirmative responses to 2 questions: “Do you happen to have in your home or garage any guns or revolvers?” and “Do any of these guns personally belong to you?”

### Measures

For this study, we examined two sets of questions from the new public safety module. The first set of questions assessed agreement with statements describing disparities in gun homicides (i.e., “Latino/a people are more likely to die from gun homicide than white people” and “Black people are more likely to die from gun homicide than white people”). The second set of questions assessed expectations of personal safety benefits associated with gun ownership or more permissive gun carrying (i.e., “Personally owning a gun will make me safer” and “I would feel safer if more people were allowed to legally carry guns”). Survey questions were asked in random order.

Responses were collected on a 5-point Likert scale ranging from “strongly agree” to “strongly disagree.” Responses were examined and then dichotomized to compare “agree” and “strongly agree” to “neither agree nor disagree,” “disagree,” and “strongly disagree.” Survey weights were applied to adjust for known selection deviations and survey nonresponse and to enable nationally representative comparisons by race/ethnicity.

### Analyses

Descriptive statistics were calculated. Logistic regression models were run to test for significant differences across race/ethnicity, stratified by gun ownership. Results are presented as percent agree with 95% confidence intervals and an alpha of 0.05. Next, using the dichotomized variables “agree” (coded as 1) and “disagree” (coded as 0), we estimated univariate logistic regression models to assess associations between specific responses and across respondent groups. Models first estimated the association between knowledge of homicide disparities and agreement with expectations of improved safety from personal gun ownership or expanded legal carrying. Then, we estimated odds of agreement with each question when comparing gun owners to non-owners, stratified by race. Finally, we estimated odds of agreement when comparing Black and Hispanic respondents to white respondents, stratified by gun ownership.

Owing to a fielding period that was punctuated by the January 6, 2021 attack on the U.S. Capitol, which may have affected partisan tensions and national feelings of security, sensitivity tests were performed to assess stability of estimates. All analyses were conducted using the *svy* command in Stata, version 16.1.^16^ The study was reviewed and approved by the Johns Hopkins Bloomberg School of Public Health IRB.

## Results

**Figure 1:**
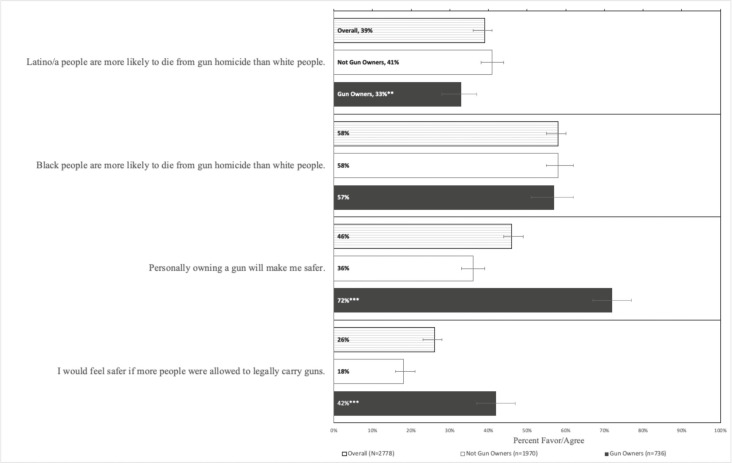
Percent of U.S. adults who agree with safety-related statements, overall and by gun ownership, 2021 Denotes significant differences in support: *p < .05, **p≤ .01, ***p ≤ .001 Reference category = Not gun owners

The survey completion rate was 78%, resulting in a total sample size of 2,778. After our oversampling, gun owners comprised 29% of the sample; 49% of respondents were white, 23% were Black, and 23% were Hispanic (results not shown). Overall, 39% of respondents agreed with the true statement of gun homicide disparities among Latinx people, and 58% agreed with the also true statement of gun homicide disparities among Black people (Fig. 1). Black respondents were the most aware of these disparities; Hispanic respondents were somewhat less aware (Appendix C, Table 1). Sixty-one percent of respondents correctly agreed with at least one of the disparities statements (95% CI: 58% - 63%); 36% responded accurately to both (95% CI: 33% - 38%) (weighted estimates; data not shown). Fewer gun owners (33%) than non-owners (41%) were aware of disparities in homicides against Latinx victims. There was no statistically significant difference in knowledge of homicide-related disparities against Black victims by gun ownership. When asked if they agreed with the statement that “personally owning a gun will make me safer,” 46% of respondents agreed (Not gun owners: 36% vs. Gun owners: 72%). When asked if they “would feel safer if more people were allowed to legally carry guns,” 26% of respondents agreed (Not gun owners: 18% vs. Gun owners: 42%) (Fig. 1).

**Table 1:** Simple logistic regression models predicting odds of agreement with safety-related statements, comparing gun owners to non-owners, stratified by race.

		White			Black			Hispanic		
		OR	95% CI	p-value	OR	95% CI	p-value	OR	95% CI	p-value
Latino/a people are more likely to die from gun homicide than white people.	Gun Owner: Non-Owner	**0.67**	**0.50 – 0.91**	**0.010**	0.95	0.56 – 1.63	0.858	0.59	0.31 – 1.15	0.125
Black people are more likely to die from gun homicide than white people.	Gun Owner: Non-Owner	0.80	0.60 – 1.08	0.146	1.70	0.91 – 3.18	0.094	0.84	0.41 – 1.69	0.617
Personally owning a gun will make me safer.	Gun Owner: Non-Owner	**7.76**	**5.71 – 10.5**	**<0.001**	**2.12**	**1.23 – 3.64**	**0.007**	**2.57**	**1.11 – 5.94**	**0.028**
I would feel safer if more people were allowed to legally carry guns.	Gun Owner: Non-Owner	**4.59**	**3.31 – 6.36**	**<0.001**	1.40	0.71 – 2.76	0.327	**2.51**	**1.23 – 5.13**	**0.011**

**Notes:**

Bold indicates: p < .05

OR = Odds Ratio

CI = Confidence Interval

**Figure 2:**
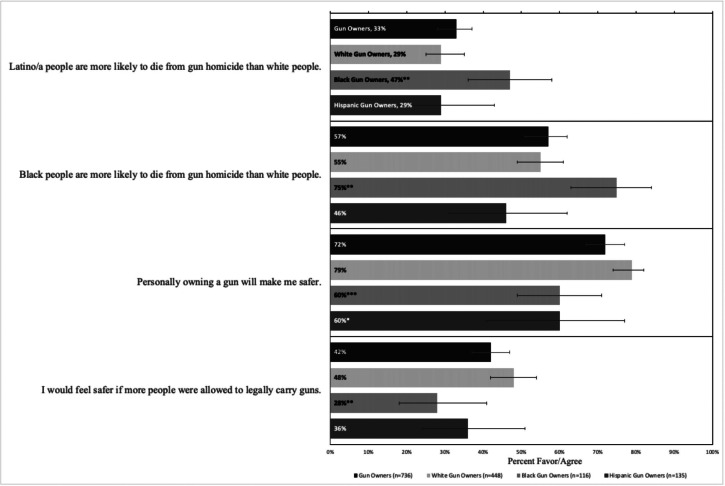
Percent of U.S. adults who agree with safety-related statements, by race/ethnicity of gun owners, 2021 Denotes significant differences in support: *p < .05, **p≤ .01, ***p ≤ .001 Reference category = Non-Hispanic White Gun Owners

Comparing non-owners of guns across racial and ethnic identities, we found no statistically significant differences in knowledge of gun homicide disparities. We also found no difference in expectations of safety associated with gun ownership or more permissive gun carrying (Appendix B, Figure 1). However, comparing Black gun owners and Hispanic gun owners to white gun owners, we found significant differences on every item. Black gun owners more frequently agreed that “Latino/a people are more likely to die from gun homicide than white people” (Black gun owner: 47% vs. white gun owner: 29%) and that “Black people are more likely to die from gun homicide than white people” (Black gun owner: 75% vs. white gun owner: 55%). Compared to white gun owners, Black and Hispanic gun owners both less frequently agreed that personal gun ownership made them safer (Black gun owners: 60%; Hispanic gun owners: 60%; white gun owners: 79%). Black gun owners also less frequently agreed that they would “feel safer if more people could legally carry guns” (Black gun owners: 28% vs. white gun owners 48%) (Fig. 2).

When the relationship between knowledge of homicide disparities and agreement with personal safety expectations was examined, we found significant associations only among white respondents. Compared to respondents who were not aware of homicide disparities, white respondents who were aware of disparities in Black and Hispanic homicide victimization had 40-42% lower odds of expecting safety to improve through personal gun ownership and 43-44% lower odds of agreeing that safety would improve through expanded legal gun carrying (results not shown). Comparing agreement by gun ownership status, we found gun owners were more likely to think that guns improve safety, but the magnitude of the association varied by race. Among white respondents, gun owners had 7.8-times higher odds of expecting improved safety from personal gun ownership (95% CI: 5.7-10.5) and 4.6-times higher odds of expecting improved safety from more widespread legal carrying (95% CI: 3.3-6.4). Among Hispanic respondents, odds of agreement were 2.6-times higher for personal gun ownership (95% CI: 1.1-5.9) and 2.5-times higher for legal carrying (95% CI: 1.2-5.1) among gun owners compared to non-owners. Among Black respondents, odds of agreement with safety gains from personal carrying were 2.1-times higher among gun owners than nonowners (95% CI: 1.2-3.6) but not significantly different on the issue of gun carrying (Table 1).

Across gun ownership strata, odds of an accurate response to the homicide disparities questions were higher among Black respondents than white respondents (OR for Latinx victimization disparities: 1.7; OR for Black victimization disparities 1.4). Compared to their white counterparts, Black non-owners had 1.5-times higher odds of agreement with Latinx victimization disparities (95% CI: 1.1-2.1), and Black gun owners had 2.1-times higher odds of agreement (95% CI: 1.2-3.6). Regarding Black victimization disparities, odds of agreement among Black gun owners were 2.5-times higher than white gun owners (95% CI: 1.4-4.5). Hispanic non-owners had 32% lower odds of agreement with Black victimization disparities (95% CI: 0.5-1.0) but were otherwise indistinct from white respondents across ownership strata (Table 2).

**Table 2:** Simple logistic regression models predicting odds of agreement with safety-related statements, comparing racial subgroups of gun owners and non-owners

		Overall			Not Gun Owners			Gun Owners		
		OR	95% CI	p-value	OR	95% CI	p-value	OR	95% CI	p-value
Latino/a people are more likely to die from gun homicide than white people.	Black: White	**1.72**	**1.31 – 2.26**	**<0.001**	**1.49**	**1.09 – 2.05**	**0.014**	**2.12**	**1.25 - 3.59**	**0.005**
	Hispanic: White	1.16	0.86 – 1.57	0.320	1.10	0.79 – 1.55	0.568	0.98	0.51 - 1.87	0.943
Black people are more likely to die from gun homicide than white people.	Black: White	**1.39**	**1.03 – 1.86**	**0.029**	1.16	0.82 – 1.63	0.394	**2.46**	**1.35 - 4.49**	**0.003**
	Hispanic: White	**0.72**	**0.54 – 0.97**	**0.032**	**0.68**	**0.49 - 0.95**	**0.024**	0.71	0.36 - 1.42	0.333
Personally owning a gun will make me safer.	Black: White	0.82	0.63 – 1.08	0.163	**1.52**	**1.09 – 2.13**	**0.014**	**0.42**	**0.25 - 0.70**	**0.001**
	Hispanic: White	**0.73**	**0.55 – 0.98**	**0.34**	1.25	0.89 – 1.76	0.201	**0.41**	**0.18 - 0.94**	**0.036**
I would feel safer if more people were allowed to legally carry guns.	Black: White	0.70	0.75 – 1.00	0.051	1.36	0.88 – 2.12	0.169	**0.42**	**0.23 - 0.76**	**0.005**
	Hispanic: White	**0.70**	**0.50 – 0.97**	**0.034**	1.12	0.72 – 1.73	0.620	0.61	0.32 – 1.17	0.139

**Notes:**

Bold indicates: p < .05

OR = Odds Ratio

CI = Confidence Interval

On issues of safety, the odds of Black non-owners agreeing that owning a gun would make them safer were 1.5-times higher (95% CI: 1.1-2.1) than white non-owners. Conversely, as compared to white gun owners, Black gun owners had 58% lower odds of agreement that gun ownership improved personal safety (95% CI: 0.25-0.70) and 58% lower odds of expected safety gains from more widespread gun carrying (95% CI: 0.23-0.76). Racial/ethnic groups of non-owners did not differ on their odds of agreement with safety improvements associated with legal gun carrying. Comparisons of Hispanic gun owners and non-owners were only different from white gun owners and non-owners on the issue of personal safety gains from owning a gun. Like Black respondents, Hispanic gun owners were 59% less likely than white gun owners to agree that gun ownership improved their personal safety (95% CI: 0.2-0.9) (Table 2).

Sensitivity tests indicated that most surveys were initiated prior to the onset of the January 6 insurrection (Appendix A). Demographic differences between pre-insurrection and during- or post-insurrection respondents were limited to age and age-related employment status (i.e., retired). In separate analyses of the pre-insurrection and during- or post-insurrection subsamples, the most notable difference in estimates was a generally higher expectation of improved safety associated with personal gun ownership and more widespread legal gun carrying among those responding during or after the insurrection. The exception to this observed trend was among the small group of during- or post-insurrection Black gun owners (n=26) (Appendix C). None of these differences in point estimates are likely to have changed inferences drawn from the overall sample compared to the pre-insurrection sample.

## Discussion

In this nationally representative survey of U.S. adults, respondents were assessed on their awareness of racial disparities in gun homicides and their expectations of improved personal safety were they to own a gun or were legal gun carrying to be expanded. We found that most Americans were generally aware of at least one example of disparities in gun homicide, with greater knowledge of over-victimization among Black Americans than white and Hispanic Americans. Black gun owners tended to be the most aware of disparities in gun violence, followed by people who did not own guns, white gun owners, and Hispanic gun owners. The odds of Black gun owners acknowledging gun homicide disparities were 2.1-2.5 times higher than white gun owners.

Compared to Americans who did not personally own a gun, gun owners of all examined races and ethnicities more frequently expected that owning a gun made them safer and that they would feel safer if more people were allowed to legally carry guns. This is unsurprising, given probable self-protection motivations for the decision to own a gun. However, our survey results indicate key differences in expectations of safety that corresponded with the racial or ethnic identity of gun owners. Relative to white gun owners, Black and Hispanic gun owners less frequently reported feeling safer because of gun ownership. Although Black non-owners were 1.5-times more likely than white non-owners to believe that gun ownership would make them safer, Black gun owners were 58% less likely than white gun owners to believe the same and 58% less likely to believe that they would be safer if more people were allowed to carry guns. In general, gun owners were less optimistic about the personal safety benefits of more permissive gun carrying than personal ownership. Only 42% of gun owners thought that more people carrying guns would improve safety. Agreement dropped to just 28% among Black gun owners.

These findings diverge somewhat from prior estimates of expected safety changes drawn from a representative survey of California residents.^17^ Compared to the California survey, we found substantially stronger expectations of safety improvements among gun owners and greater differences between gun owners’ assessments of others’ actions (i.e., feelings of safety associated with other people owning or carrying guns) compared to their own actions (i.e., feelings of safety associated with personal gun ownership). This finding is consistent with research on other assessments of skills with potential for injury, such as driving.^18^ With regard to gun safety, divergence from the California survey may be explained by different social contexts associated with California’s restrictive gun policies, relative to the U.S. overall, or by subtle differences in the two surveys’ questions about safety. The California survey asked about expectations of improved safety of the home or neighborhood associated with one’s own or others’ in-home gun possession, respectively; our survey asked about anticipated benefits to personal safety resulting from ownership or presumably public gun carrying by others. Whether gun owners hold different beliefs about personal safety versus safety of the home environment may vary by an individual’s role in the home and by sources of the perceived threat.^19^

The results presented here suggest persistent safety concerns among many Black Americans, despite (in some cases) having personally acquired a gun, which is usually motivated by self-protection.^20^ The subsequently lower feelings of safety among Black compared to white gun owners may be interpretated as indication of persistent differences in risk for violent crime victimization or may be evidence of knowledge and beliefs that are protective against other potential sources of gun-related injury.^21^ The sources of threat underlying these feelings may endure or may evolve over time, potentially to include unique concerns related to how one is perceived as a gun owner. For Black men in particular, these concerns may include the potential for violence to be used against them in acts motivated by racist presumptions of criminality and expectations of restricted access to legal gun ownership.^22^

Our study suggests that homicide risk alone does not explain motivations for gun purchase, nor does it sufficiently explain racially divergent feelings of safety following gun acquisition. Nonfatal violence, including injuries and various forms of threatened violence, may be additionally influential threats to safety. These findings may be informative to the interpretation of prior research, which suggested that Black Americans, across gun ownership status, tend to favor more restrictive gun policies, police reforms, and efforts to expand community-based gun violence prevention.^23^ Perceptions of personal safety and personal experiences with public safety interventions may influence support for policy reform as well as motivations for gun acquisition. Additional research is needed to understand drivers of differences in perceived safety among gun owners.

Some limitations should be considered. First, this was a cross-sectional analysis, which limited our ability to infer a temporal relationship between safety concerns and gun ownership. In particular, gun owners’ expectations of safety prior to gun ownership should not be assumed, nor should the future gun ownership intentions of current non-owners. Still, evidence of some dissatisfaction with safety after gun ownership is clear and appears to be greatest among Black gun owners. Interpretation of the views expressed by respondents identified as Hispanic may have been confounded by variably racialized co-identities as Black, Brown, or white. Other factors, such as gender, political affiliation, worldview, or urban/rural residential status, may also contribute to perceptions of safety. Sample size limited our capacity to explore these potential co-contributors while still maintaining the intersectional identities of original interest. As in any survey, our findings may be affected by sampling bias and question framing. NORC’s use of probability-based sampling helps to minimize the sampling threat. Other framing may yield different results. Finally, the January 6, 2021 attack on the U.S. Capitol coincided with the fielding of this survey and may have affected respondents’ feelings of safety or beliefs about guns in potentially racially disparate ways. We conducted a sensitivity analysis to assess whether this event was likely to have affected our main study findings. Despite some differences in point estimates, we found comparative interpretations to be largely unaffected.

## Implications

In the context of the 2022 Supreme Court ruling in *New York State Rifle & Pistol Association v. Bruen* ^24^ and an imminent movement nationally toward the conditions described in this survey (specifically, more people being allowed to legally carry guns and risk for further violent crime disparities^25^), these differences in American views and experiences of gun-related safety have important implications. First, our findings suggest that most people do not agree that more widespread legal gun carrying will make them feel safer. This challenges the notion of more “good guys with guns” as a protective American ideal. Rather, our findings suggest that majorities of gun owners and non-owners, alike, have reservations about the personal safety gains of more widespread public gun carrying. This is particularly true among Black Americans. At the same time, relatively more Americans (though notably still less than half) believe that owning a gun will improve their personal safety.

If acted on, these two conditions — concerns about the safety consequences of more public gun carrying but a relatively stronger belief in the personal protectiveness of gun ownership — may provoke a self-perpetuating sense of insecurity in the U.S. Specifically, safety concerns driving protection-motivated gun acquisition may be further increased by subsequently more gun ownership within a permissive gun carrying policy context. Moreover, our findings suggest that expectations of safety associated with gun ownership and public carrying among American gun owners are racially unequal. Given this, targeted public health messaging from health providers and trusted messengers to gun owners and non-owners is needed and should include evidence-informed, actionable steps to protect personal safety. Healthcare providers can similarly counsel individuals when personal safety concerns are assessed. Such messages may include the promotion of safe gun storage, responsible gun commerce, and information about mechanisms for alternative, temporary storage in times of crisis. If widely implemented, such actions may help to reduce unequal downstream risks related to homicide and other threats to safety across the life course. Additionally, in this critical time in which a new legal landscape remains uncertain, vocal public support for policies that have proven popular^26^ and could reduce gun violence more broadly, such as purchasing permits^27^ and prohibitions on gun carrying in sensitive areas,^28^ is urgently needed to improve the equity of health and safety in the United States.

## Figures and Tables

**Appendix A:**
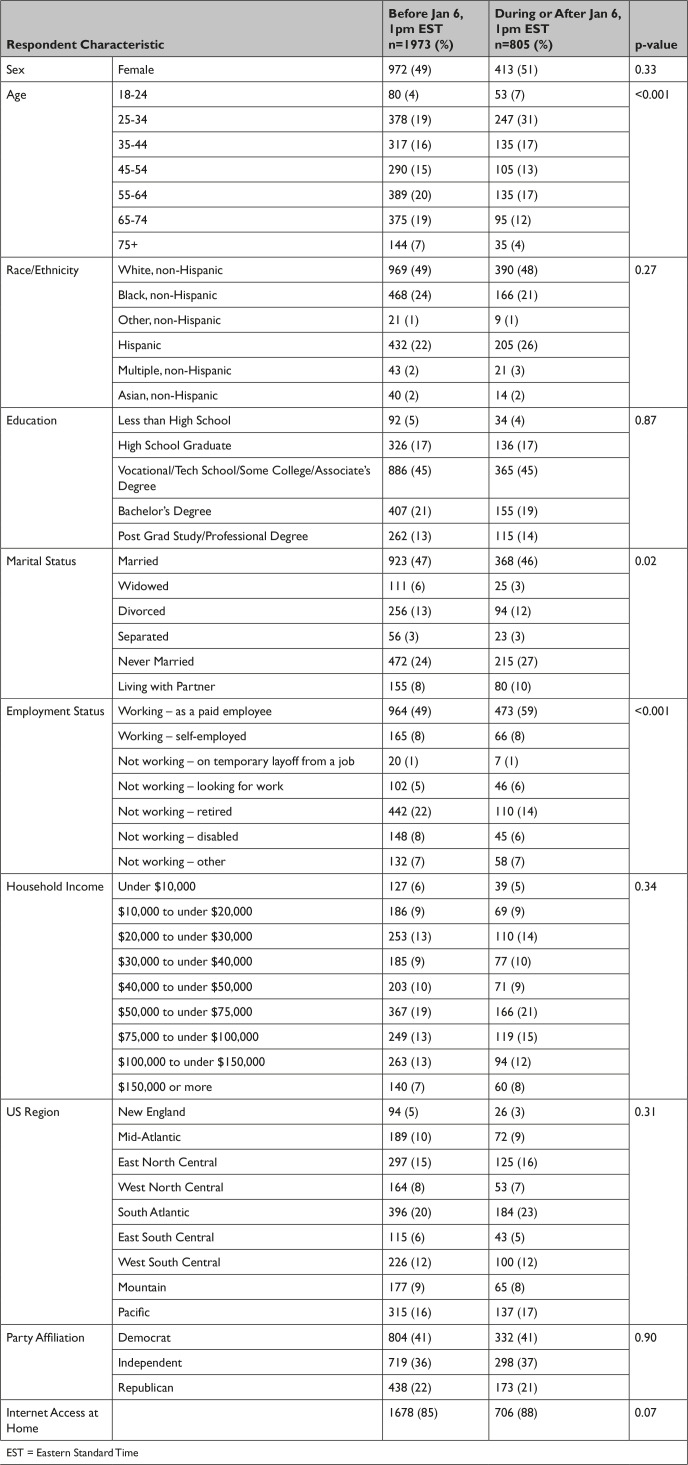
Demographic characteristics of pre- and during-/post-insurrection respondents (unweighted)

**Appendix B, Figure 1:**
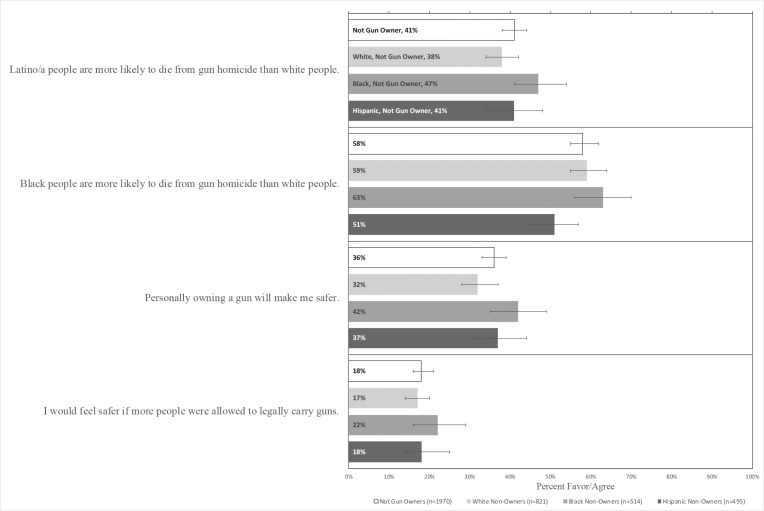
Percent of U.S. adults who agree with safety-related statements, by race/ethnicity of people who were not gun owners, 2021 Reference Category = Non-Hispanic White, Not Gun Owners

**Appendix C. Table 2:**
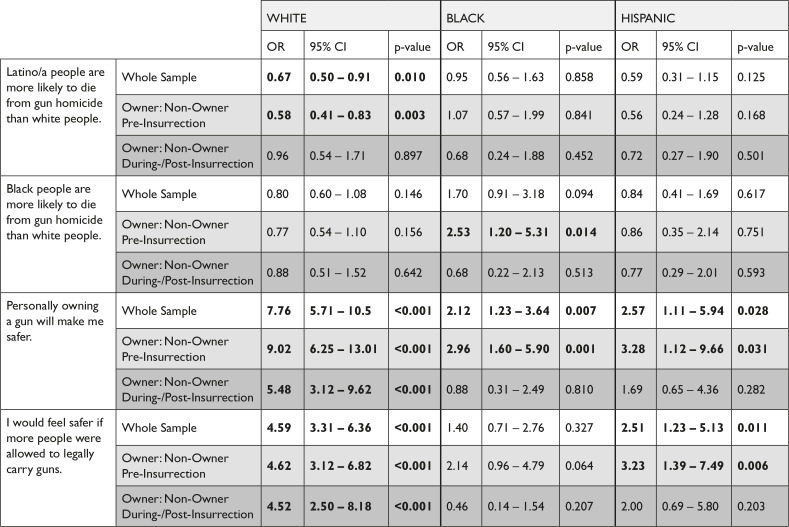
Simple logistic regression models predicting odds of agreement with safety-related statements, comparing gun owners to non-owners, stratified by race and time of survey completion (weighted)

**Notes:**

Bold indicates: p < .05

OR = Odds Ratio

CI = Confidence Interval

**Appendix C. Table 1:**
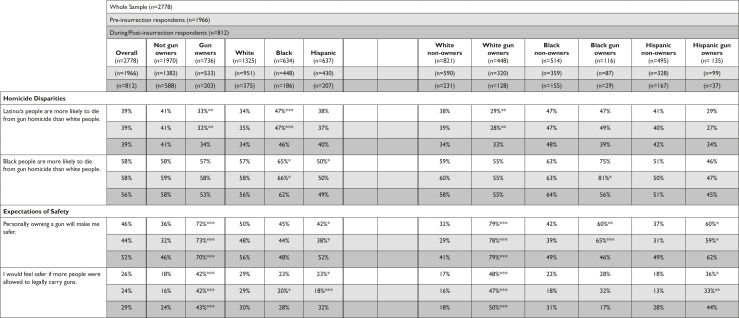
Percent of U.S. adults who agree with safety-related statements, overall and by race and gun ownership, stratified by time of survey completion (weighted)

Denotes significant differences in support: *p < .05, **p≤ .01, ***p ≤ .001

Reference category = Gun owners or non-owners within same race/ethnicity category

**Appendix C. Table 3:**
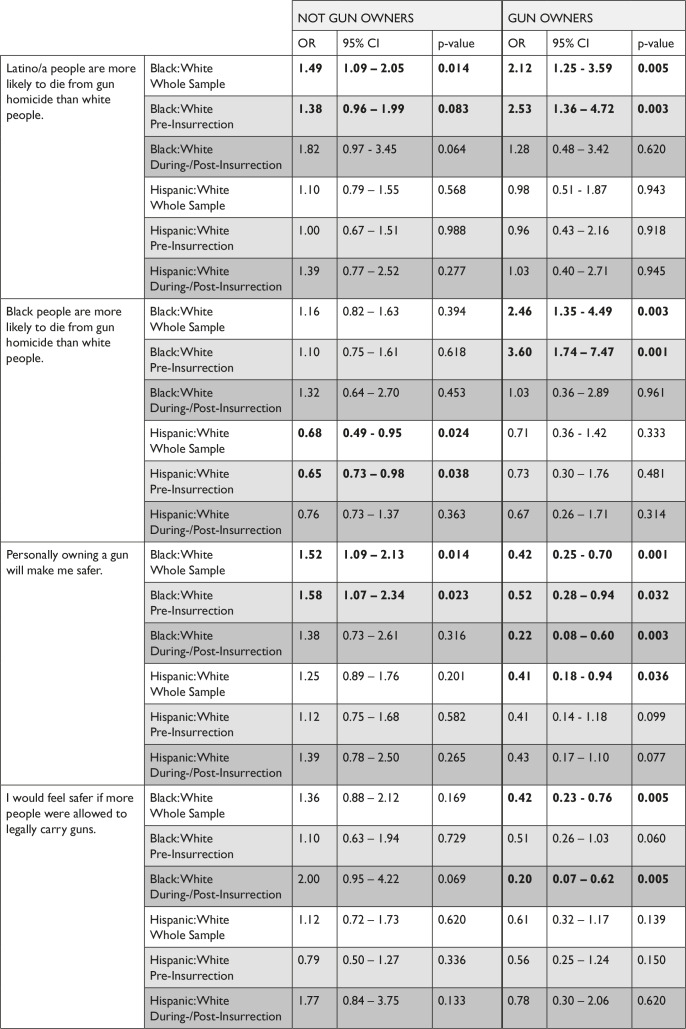
Simple logistic regression models predicting odds of agreement with gun-related safety statements among U.S. adults, by race/ethnicity, stratified by gun ownership and time of survey completion (weighted)

**Notes:**

Bold indicates: p < .05

OR= Odds Ratio CI = Confidence Interval

## Note

Ms. Ward reports grants from National Institute of Child Health and Human Development, during the conduct of the study. Dr. Crifasi reports grants from Bloomberg American Health Initiative, during the conduct of the study, and the Centers for Disease Control and Prevention, Robert Wood Johnson Foundation, New Venture Fund, and Joyce Foundation, outside the submitted work. The other authors have no conflicts to disclose.

## References

[1] S.R. Kegler , T.R. Simon , M.L. Zwald , et al., “Vital Signs: Changes in Firearm Homicide and Suicide Rates — United States, 2019–2020,” Morbidity and Mortality Weekly Report 71, no. 19 (2022): 656–663, doi:10.15585/mmwr.mm7119e1. 35550497 PMC9098246

[2] *Id.*; R. Thebault and D. Rindler, “Shootings Never Stopped During the Pandemic: 2020 was the Deadliest Gun Violence Year in Decades,” *Washington Post*, March 23, 2021, *available at* <https://www.washingtonpost.com/nation/2021/03/23/2020-shootings/> (last visited Feb. 10, 2023); R. Rosenfeld and E. Lopez , “Pandemic, Social Unrest, and Crime in U.S. Cities,” Federal Sentencing Reporter 33, no. 1-2 (2020): 72–82, doi:10.1525/fsr.2020.33.1-2.72.

[3] Kegler, *supra* note 1.

[4] F. Edwards , H. Lee , and M. Esposito , “Risk of Being Killed by Police use of Force in the United States by Age, Race–Ethnicity, and Sex,” Proceedings of National Academy of Science 116, no. 34 (2019): 16793–16798, doi:10.1073/pnas.1821204116. PMC6708348 31383756

[5] R.F. Wilson , G. Liu , B.H. Lyons , et al., “Surveillance for Violent Deaths — National Violent Death Reporting System, 42 States, the District of Columbia, and Puerto Rico, 2019,” Morbidity and Mortality Weekly Report 71, no. 6 (2022): 44, *available at* < 10.15585/mmwr.ss7103a1> (last visited April 3, 2023). PMC9129903 35588398

[6] J. Gramlich, “Safety Concerns were Top of Mind for Many Black Americans before Buffalo Shooting,” Pew Research Center, *available at* <https://www.pewresearch.org/fact-tank/2022/05/20/safety-concerns-were-top-of-mind-for-many-black-americans-before-buffalo-shooting/> (last visited Feb. 10, 2023).

[7] C.K. Crifasi , J.A. Ward , E.E. McGinty , D.W. Webster , and C.L. Barry , “Gun Purchasing Behaviours during the Initial Phase of the COVID-19 Pandemic, March to Mid-July 2020,” International Review of Psychiatry 33 (2021): 593–597, doi:10.1080/09540261.2021.1901669; M. Miller and D. Azrael, “Who Bought Guns during the Pandemic? Previewing New Survey Data,” The Joyce Foundation, YouTube, Published June 2, 2021, *available at* <https://www.youtube.com/watch?v=mXD0m77R3mY> (last visited Feb. 10, 2023). PMC11526816 34167429

[8] Miller, *supra* note 7.

[9] R. Pallin , S.A. Spitzer , M.L. Ranney , M.E. Betz , and G.J. Wintemute , “Preventing Firearm-Related Death and Injury,” Annals of Internal Medicine 170, no. 11 (2019): ITC81–ITC96, doi:10.7326/AITC201906040; M.L. Doucette, J.A. Ward, A.D. McCourt, D. Webster, and C.K. Crifasi, “Officer-involved shootings and concealed carry weapons permitting laws: Analysis of Gun Violence Archive data, 2014–2020,” *Journal of Urban Health* 99, no. 3 (2022): 373-384, doi:10.1007/s11524-022-00627-5. 35536393 PMC9187822

[10] J.M. Pierre , “The Psychology of Guns: Risk, Fear, and Motivated Reasoning,” Palgrave Communications 5, no. 1 (2019): 1–7, doi:10.1057/s41599-019-0373-z; W. Hauser and G. Kleck, “Guns and Fear: A One-Way Street?” *Crime & Delinquency* 59, no. 2 (2013): 271-291, doi:10.1177/0011128712462307; J.P. Schleimer, G.J. Wintemute, and N. Kravitz-Wirtz, “Firearm Ownership and Perceived Risk of Personal Firearm Injury,” *Injury Prevention* 27, no. 3 (2021): 277-279, doi:10.1136/injuryprev-2020-043869; R. Pallin, G.J. Wintemute, and N. Kravitz-Wirtz, “‘What Does it Depend On?’: Perceptions of Safety Related to Firearms in Homes and Neighborhoods,” *PLoS ONE* 16, no. 12 (2021): e0261038, doi:10.1371/journal.pone.0261038. PMC8716056 34965246

[11] E.R. Morgan , A. Rowhani-Rahbar , D. Azrael , and M. Miller , “Public Perceptions of Firearm- and Non–Firearm-Related Violent Death in the United States: A National Study,” Annals of Internal Medicine 169, no. 10 (2018): 734–737, doi:10.7326/M18-1533; M.C. Gearhart, K.A. Berg, C. Jones, and S.D. Johnson, “Fear of Crime, Racial Bias, and Gun Ownership,” *Health & Social Work* 44, no. 4 (2019): 241-248, doi:10.1093/hsw/hlz025; A.C. Thomas, B.J. Siry-Bove, L.M. Barnard, et al., “A Qualitative Study on Diverse Perspectives and Identities of Firearm Owners,” *Injury Prevention* 28, no. 5 (2022), doi:10.1136/injuryprev-2022-044522; J.T. Pickett, A. Graham, and F.T. Cullen, “The American Racial Divide in Fear of the Police,” *Criminology* 60, no. 2 (2022): 291-320, doi:10.1111/1745-9125.12298. 30383115

[12] M. Miller , W. Zhang , and D. Azrael , “Firearm Purchasing during the COVID-19 Pandemic: Results from the 2021 National Firearms Survey,” Annals of Internal Medicine 175 (2022): 219–225, doi:10.7326/M21-3423. 34928699 PMC8697522

[13] C.L. Barry , E.E. McGinty , J.S. Vernick , and D.W. Webster , “After Newtown — Public Opinion on Gun Policy and Mental Illness,” New England Journal of Medicine 368, no. 12 (2013): 1077–1081, doi:10.1056/NEJMp1300512; C.L. Barry, E.E. McGinty, J.S. Vernick, and D.W. Webster, “Two Years after Newtown — Public Opinion on Gun Policy Revisited,” *Preventive Medicine* 79 (2015): 55-58, doi:10.1016/j.ypmed.2015.05.007; C.L. Barry, D.W. Webster, E. Stone, C.K. Crifasi, J.S. Vernick, and E.E. McGinty, “Public Support for Gun Violence Prevention Policies among Gun Owners and Non–Gun Owners in 2017,” *American Journal of Public Health* 108, no. 7 (2018): 878-891, doi:10.2105/AJPH.2018.304432; C.L. Barry, E.M. Stone, C.K. Crifasi, J.S. Vernick, D.W. Webster, and E.E. McGinty, “Trends in Public Opinion on US Gun Laws: Majorities of Gun Owners and Non–Gun Owners Support a Range of Measures,” *Health Affairs (Millwood)* 38, no. 10 (2019): 1727-1734, doi:10.1377/hlthaff.2019.00576. 23356490

[14] J.A. Ward , E.E. McGinty , T. Hudson , et al., “Reimagining Public Safety: Public Opinion on Police Reform and Gun Violence Prevention by Race and Gun Ownership in the United States,” Prevention Medicine 165 (2022). 10.1016/j.ypmed.2022.107180 PMC9722519 35933003

[15] NORC, Technical Overview of the AmeriSpeak Panel NORC’s Probability-Based Household Panel (University of Chicago, 2022): 1–7, *available at* <https://amerispeak.norc.org/Documents/Research/AmeriSpeak%20Technical%20Overview%202019%2002%2018.pdf> (last visited Feb. 13, 2023).

[16] StataCorp. Release 16.1, published online 2020.

[17] Pallin, *supra* note 10.

[18] M.M. Roy and M.J. Liersch , “I am a Better Driver than you Think: Examining Self-Enhancement for Driving Ability,” Journal of Applied Social Psychology 43, no. 8 (2013): 10.1111/jasp.12117, doi:10.1111/jasp.12117. PMC3835346 24273339

[19] Pierre, *supra* note 10; Schleimer, *supra* note 10.

[20] W. Stroebe , N.P. Leander , and A.W. Kruglanski , “Is it a Dangerous World Out There? The Motivational Bases of American Gun Ownership,” Personality & Social Psychology Bulletin 43, no. 8 (2017): 1071–1085, doi:10.1177/0146167217703952. 28903713

[21] Pallin, *supra* note 9; Doucette *supra* note 9.

[22] J. Carlson , “Legally Armed but Presumed Dangerous: An Intersectional Analysis of Gun Carry Licensing as a Racial/Gender Degradation Ceremony,” Gender & Society 32, no. 2 (2018): 204–227, doi:10.1177/0891243217745862; A. Stroud, “Good Guys with Guns: Hegemonic Masculinity and Concealed Handguns,” *Gender & Society* 26, no. 2 (2012): 216-238, doi:10.1177/0891243211434612; G.D. Higginbotham, D.O. Sears, and L. Goldstein, “When an Irresistible Prejudice Meets Immovable Politics: Black Legal Gun Ownership Undermines Racially Resentful White Americans’ Gun Rights Advocacy,” *Journal of Experimental Psychology General* (2022), doi:10.1037/xge0001275. 36006732

[23] C.K. Crifasi , J.A. Ward , E.E. McGinty , D.W. Webster , and C.L. Barry , “Public Opinion on Gun Policy by Race and Gun Ownership Status,” Preventive Medicine 149 (2021): 106607, doi:10.1016/j.ypmed.2021.106607; Ward, *supra* note 14. 33984373 PMC8273872

[24] *New York State Rifle & Pistol Association, Inc., et al., Petitioners v. Kevin P. Bruen, in His Official Capacity as Superintendent of New York State Police, et Al.* 200 321, 337 (Supreme Court of the United States 2022), *available at* <https://www.supremecourt.gov/opinions/21pdf/20-843_7j80.pdf> (last visited Feb. 13, 2023).

[25] E. Vicens , S. Lavender , R. Polan , and K. Wright , Brief of Amici Curiae Social Scientists and Public Health Researchers in Support of Respondents. Cleary Gottlieb Steen & Hamilton LLP; 2021:54, *available at* <https://www.supremecourt.gov/DocketPDF/20/20-843/193173/20210921125521825_BRIEF%20OF%20AMICI%20CURIAE%20SOCIAL%20SCIENTISTS%20AND%20PUBLIC%20HEALTH%20RESEARCHERS%20IN%20SUPPORT%20OF%20RESPONDENTS.pdf> (last visited Feb. 13, 2023).

[26] E.M. Stone , C.K. Crifasi , J.A. Ward , et al., “National Support for Gun Policies among U.S. Adults in 2019 and 2021,” Prevtive Medicine 165 (2022), doi:10.1016/j.ypmed.2022.107314; C.K. Crifasi, J.A. Ward, E.E. McGinty, C.L. Barry, and D.W. Webster, “Public Opinion on Laws Regulating Public Gun Carrying,” *Preventive Medicine* 159 (2022): 107067, doi:10.1016/j.ypmed.2022.107067. PMC11578283 35460721

[27] J. Mascia, “The Supreme Court’s Gun Decision Could Open this Policy up to Court Challenges,” *The Trace*, *available at* <https://www.thetrace.org/2022/07/supreme-court-permit-to-purchase-laws/> (last visited Feb. 13, 2023).

[28] D.A.H. Miller, “The Next Front in the Fight Over Guns,” *Washington Post*, *available at* <https://www.washingtonpost.com/outlook/2022/07/01/bruen-guns-rights-carry-sensitive-places/> (last visited Feb. 13, 2023).

